# Polystyrene Accelerates Aging Related-Gut Microbiome Dysbiosis and -Metabolites in Old-Aged Mouse

**DOI:** 10.4014/jmb.2504.04016

**Published:** 2025-08-07

**Authors:** Hyun Hwangbo, Eun-Ju Kim, Gi-Young Kim, Sun-Young Hwang, Mee-Hyun Lee, Yung Hyun Choi

**Affiliations:** 1 Department of Biochemistry, Dong-eui University College of Korean Medicine, Busan 47227, Republic of Korea; 2 Basic Research Laboratory for the Regulation of Microplastic-Mediated Diseases and Anti-Aging Research Center, Dong-eui University, Busan 47227, Republic of Korea; 3 College of Korean Medicine, Dongshin University, Naju 58245, Republic of Korea; 4 Department of Marine Life Science, Jeju National University, Jeju 63243, Republic of Korea

**Keywords:** Polystyrene, gut microbiota, metabolite, aging, 16S rDNA sequencing

## Abstract

Microplastics, particularly polystyrene (PS), are ubiquitous environmental contaminants and concerns about their potential detrimental effects on human health are increasing. Emerging evidence suggests that microplastics may disrupt the gut microbiota, a critical ecosystem involved in regulating host metabolism, immunity, and aging processes. However, the specific effects of PS on the gut microbiota composition and its potential role in modulating aging are yet to be fully elucidated. In this study, we aimed to investigate the effects of PS exposure on gut microbiota dysbiosis and its potential role in the acceleration of aging. Gut microbiota composition was assessed using 16S rDNA sequencing, while fecal metabolites were analyzed using gas chromatography-mass spectrometry. Exposure to PS resulted in a significant reduction in the abundance of beneficial microbiota, including *Blautia*. In contrast, there was an increase in the relative abundance of potentially harmful taxa, such as *Lachnospiraceae UCG-001*, and *Candidatus Arthromitus*. Metabolomic analysis revealed elevated levels of several metabolites associated with stress responses and altered host metabolism, including alanine, serine, tryptophan, 5-aminovaleric acid, thymine, threonine, methionine, and benzoic acid. These findings demonstrate that PS exposure in aged mice exacerbated gut microbiome dysbiosis and altered key metabolic markers associated with aging, suggesting an increased vulnerability to age-related diseases as a consequence of microplastic exposure.

## Introduction

Microplastics (MPs) have experienced a sustained increase in global plastic production in recent years [1]. MPs, including polypropylene (PP), polyethylene (PE), polyethylene terephthalate (PET) and polystyrene (PS), are commonly found in food containers and packaging materials. Among them, PS is one of the most widely used polymers in the production of food containers and packaging [2]. The rapid increase in the use of PS has led to its accumulation in the human body through various environmental matrices such as soil, water, beverages, and air. Consequently, significant health concerns have been raised about human exposure to MPs through ingestion and inhalation. Increasing evidence suggests that MPs interact with biological systems and may contribute to conditions such as DNA damage, organ dysfunction, metabolic disorders, and immune dysregulation [3].

The gut microbiome plays a critical role in human health, and dysbiosis has been linked to various diseases such as inflammatory bowel diseases [4], aging [5] anxiety and depression [6]. The intake of MPs has been shown to promote gut microbiome dysbiosis [7], which has been implicated in the development of obesity [2, 8], metabolic syndrome [9, 10], liver injury [11] and cognitive impairment [12, 13]. The gut microbiota, often referred to as the “second brain” due to its extensive influence on host physiology, constitutes the largest microbial reservoir in the human body. Increasing evidence suggests that alterations in the gut microbiota contribute to the development of various neurological and psychiatric disorders, particularly those associated with aging, through the bidirectional gut microbiota-brain axis [12]. MP-induced dysbiosis of the gut microbiota has been implicated in the development of neuropsychiatric disorders, including autism spectrum disorder [14], depression [15], Alzheimer’s disease, and Parkinson’s disease [16]. While many studies have examined the effects of MPs on disease progression by inducing pathological conditions and assessing gut microbiome dysbiosis, this study aimed to explore whether MP accelerates aging by administering it to aged mice and evaluating the resulting alterations in the gut microbiota. The incidence of these diseases increases with aging, so the relationship between MPs and age-related diseases should be investigated.

Age may influence changes in gut microbiota associated with disease. This suggests that gut microbiome may accelerate aging. Many studies have reported that the gut microbiota changes with host aging. Moreover, the gut microbiome is emerging as a key player in understanding several aging-related disorder such as Parkinson disease and Alzheimer’s disease [17, 18]. Previous studies have reported that the administration of MP leads to an increase in gut microbiota [19] such as *Helicobacter* and *Clostridia UCG-014*, which were also evaluated in mouse models of Alzheimer’s disease [20]. However, most of these studies have utilized 4 weeks old mice, which may not be sufficiently aged to accurately assess the relationships between MP administration, aging, and gut microbiota alterations. Therefore, we administered MPs to 12 months old mice to investigate the association between gut microbiota alterations and aging. During aging, the composition of the microbiome changes and its abundance decrease as gut microbiome imbalances occur [21]. It is closely related to brain health and aging. It is necessary to determine whether aging is accelerated due to gut microbiome dysbiosis. However, few studies have described the relationship between PS, aging, and gut microbiome dysbiosis.

Therefore, the aim of this study was to investigate how gut microbiota composition changes following PS administration in aged mice, and whether these changes contribute to the acceleration of aging. This study focused on the examining the influence of the gut microbiome community and how changes in metabolites during PS intake accelerates aging.

## Materials and Methods

### Animal Study

In this study, 6-week-old and 12-month-old C57BL/6 mice (Koatech Inc., Republic of Korea) were used. The animals were acclimatized for one week under controlled conditions with a temperature of 21 ± 2°C, humidity of 50 ± 5%, and a 12-h light/dark cycle. After the acclimatization period, only healthy animals were selected for the experiments. The mice were randomly divided into three groups: Young Normal group (YN), Old Normal group (ON), PS-NP-treated of old group (PS). PS-NP was administered orally at a dose of 50 mg/kg, five times per week, for a total of four weeks. Fluoresbrite YG Microspheres 0.1 μm (Polysciences, Inc., Cat. 170150) were used as the polystyrene nanoparticles in this study. Fecal samples were collected at three time points, specifically during the 3rd and 4th weeks of the experiment, and immediately before the end of the study. The collected samples were stored at -80°C until further analysis. At the end of the experimental period, the animals were euthanized, and major organs, including the heart, liver, spleen, and kidneys, were excised and weighed. All animal experiments were conducted in accordance with protocols approved by the Institutional Animal Care and Use Committee (IACUC) of Dong-Eui University (Approval No. A2024-004).

### Serum Biochemical Analysis

Serum biochemical analysis was performed to measure the levels of aspartate aminotransferase (AST), alanine aminotransferase (ALT), creatinine, and glucose. Blood samples were collected and centrifuged to separate the serum. The levels of AST and ALT were determined using commercially available enzymatic assay kits, following the manufacturer's instructions. Serum creatinine levels were measured using the Jaffe method, and glucose levels were quantified with the enzymatic hexokinase method. Absorbance values were recorded using a spectrophotometer, and concentrations were calculated from standard curves for each analyte.

### DNA Extraction from Fecal Samples and Sequencing

DNA extraction from 100 mg of fecal was carried out using the AccuFAST automation system (AccuGene Inc., Republic of Korea) following the manufacturer’s protocol. For MiSeq sequencing, bacterial 16S rRNA genes were amplified using primers targeting the V3-V4 hypervariable region. The amplification was performed through 25 PCR cycles using KAPA HiFi HotStart ReadyMix (Roche Sequencing, USA). The resulting PCR products (~250 bp) were purified using HiAccuBeads (AccuGene Inc.) for next-generation sequencing (NGS) library preparation. The purified amplicon libraries were pooled in equimolar concentrations and sequenced on an Illumina MiSeq system using the MiSeq Reagent Kit v2 (500 cycles, Illumina, USA).

### Sample Library Normalization

Raw sequencing data were processed to correct amplicon errors and denoise sequences, enabling the identification of exact amplicon sequence variants (ASVs) using DADA2 v1.16. The SILVA release 138 rRNA reference database was employed to build a Naïve Bayes classifier, which was then used to classify ASVs generated through DADA2. Microbiome bioinformatics analyses were conducted using the Quantitative Insights Into Microbial Ecology (QIIME2-2022.2) software package. The sequences obtained from the DADA2 pipeline underwent quality filtering, denoising, merging, and chimera removal. The ASVs derived from 16S rRNA gene amplicon sequencing were processed using Phylogenetic Investigation of Communities by Reconstruction of Unobserved States 2 (PICRUSt) software to predict functional genes associated with the classified gut microbiota members, based on reference-based ASV selection against the SILVA release 138 database. For alpha diversity analysis, operational taxonomic units (OTUs) with a 97% similarity threshold were utilized, assessing diversity within individual samples through Chao 1, Observed features, Shannon, and Simpson indices. Beta diversity analysis was conducted to compare bacterial community structures across groups using Bray-Curtis and Jaccard distance metrics.

### Fecal Untargeted Metabolomics Analysis and Data Processing

Fecal samples (100 mg) were individually collected, and each homogenate was thoroughly mixed with 500 μl of methanol. The samples were then incubated on ice for 5 min before being centrifuged at 13,000 rpm, 4°C for 15 min. The resulting supernatant was subjected to analysis using a QP 2020 Gas Chromatography-Mass Spectrometry (GC-MS) system (Shimadzu, Japan). The GC-MS conditions followed those described in a previous study [22]. Metabolite annotation was carried out using the MS-DIAL platform, with a mass range of 50-500 m/z set according to the acquisition method. For marker detection, a minimum amplitude threshold of 1000 was applied, with a scan level of 3 and an average peak width of 20 scans. Data points were processed using a linear weighted average for smoothing. Peak deconvolution was performed with a resolution of 0.5, as lower values could result in signal noise being misinterpreted as peaks, while higher values might limit the number of resolved chromatographic peaks. Metabolite identification was based on retention indices calculated using the Kovats method, which involved the injection of an alkane standard mixture, and was further validated by matching characteristic m/z fragmentation patterns with those of authentic standard compounds using the Kovats RI library. All features extracted from MS-DIAL were used to construct chemometric models with SIMCA version 18.0 software (Umetrics, Sartorius Stedim Biotech AS, Sweden).

### Statistical Analysis

For all the statistical data analyses, Graphpad prism (version 8.0.1, USA) were performed with analysis of variance (ANOVA) of Benjamini tests and all data are expressed as the mean ± standard deviation. Statistical significance was set at *p* < 0.05. Multiple testing was corrected using the positive false discovery rate (FDR) by computing q-values after the ANOVA test. We performed several statistical analyses using Metaboanalyst 6.0. Partial least squares discriminant analysis (PLS-DA) was performed to identify differences between metabolite profiles of YN, ON and PS groups. The VIP (Variable importance in projection) > 1 and *p*-value < 0.05 were taken to identify the features that significantly differentiated between YN, ON and PS groups, and the fold change ratio for each feature was obtained.

## Results

### Measurement of Physiologic Index Values in the YN, ON and PS Groups

**Fig. 1:**
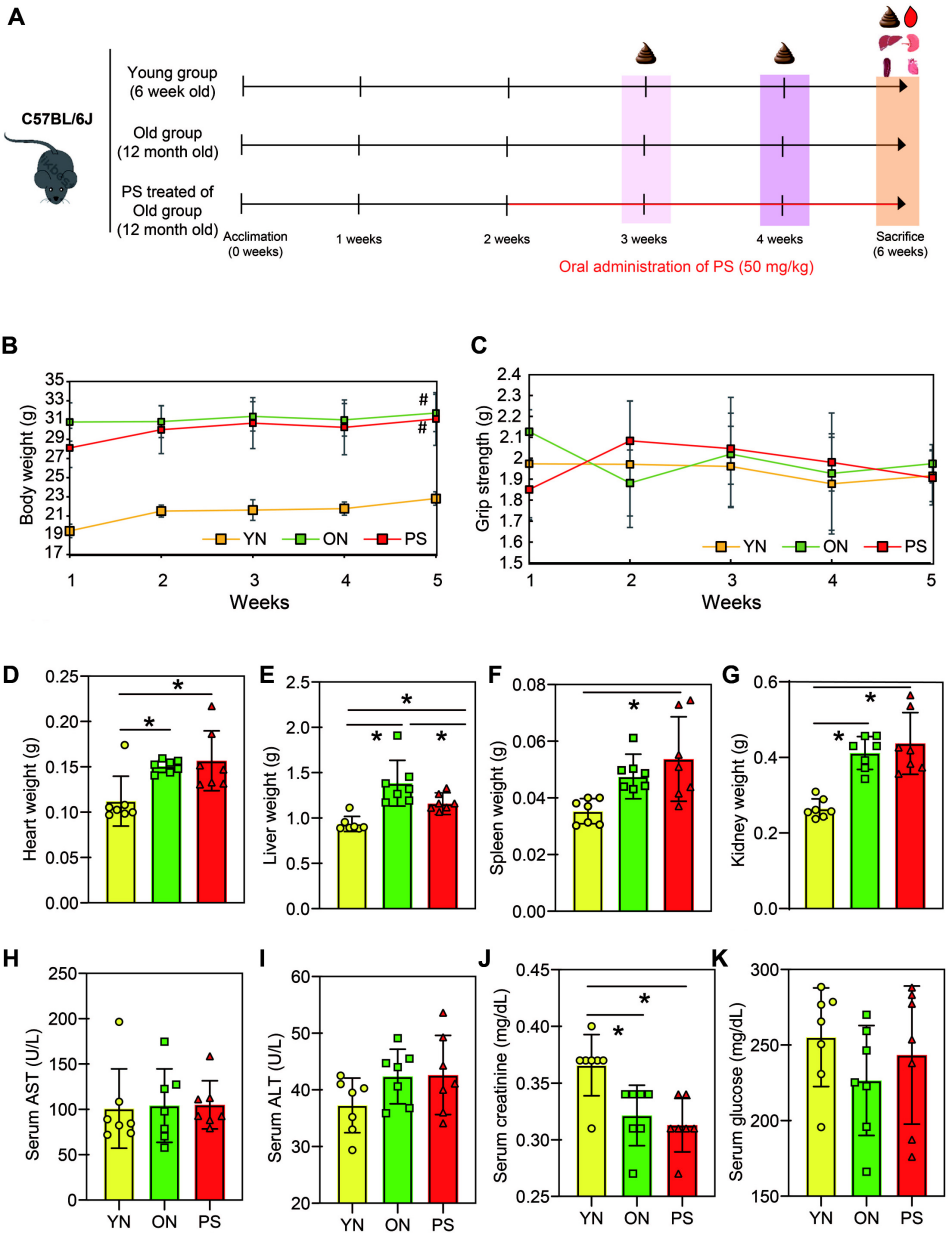
Measurement of physiologic index on YN, ON and PS group. (**A**) Schematic of experiment design. (**B**) Body weight calculated at 5 weeks; Data were compared using one-way ANOVA with a Benjamini and Hochberg multiple comparison test for individual time points. (**C**) Comparison of the average grip strength of YN, ON and PS group. (**D-G**) Histological weight. (**H-K**) Measurement of serum biochemical indices; AST, ALT, creatinine and glucose levels. Data are expressed as mean ± SD. **** *p* < 0.0001, * *p* < 0.05, N.S : not significant.

Fig. 1A provides a comprehensive overview of the five-week experimental period. The young normal (YN) group consistently maintained a lower body weight compared to the old normal (ON) and PS-treated (PS) groups. Notably, the PS group exhibited a gradual increase in body weight, similar to the ON group, suggesting that PS exposure may contribute to age-related weight gain (Fig. 1B). Grip strength measurements showed fluctuations across the weeks (Fig. 1C). While the YN group exhibited relatively stable grip strength over time, the PS group showed an initial increase followed by a gradual decline, aligning with the tendency observed in the ON group. Although no significant differences were observed among the groups, PS administration may contribute to age-related muscle function decline. The bar graphs illustrate the impact of PS administration on various metabolic markers (Fig. 1D-1K). The PS group showed significantly higher levels of spleen and kidney weight than the YN group, comparable to the ON group (Fig. 1 and 1G). The PS group showed significantly lower serum creatinine levels than the YN and ON groups (Fig. 1J).

### Effects of PS Treatment on Gut Microbial Community Composition and Diversity

**Fig. 2:**
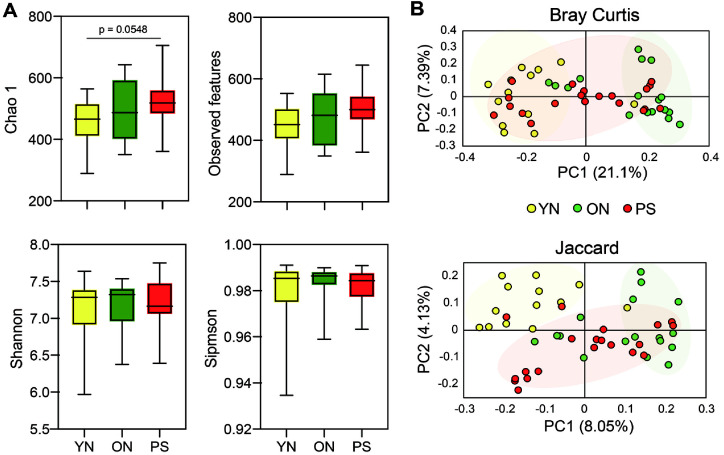
Effects of PS intake on the composition of gut microbiota. Analysis of alpha diversity in control group with young and old mouse compared with treated PS groups (**A**). Principal coordinate of (**B**) Bray curtis and Jaccard distance.

To intake the effect of PS plastics exposure on the gut microbiota, we analyzed 16S rDNA sequencing on the fecal DNA from the young and old and PS treated groups. In Fig. 2A, there were no significant differences in Observed features, Shannon and Simpson index values among the three groups. In Chao 1 index, the PS group tended to be higher than the ON group, and p value was 0.0548, which was very close to 0.05. As for beta diversity, the differences in the bacterial composition among the three groups were evaluated using principal coordinate analysis (Fig. 2B). In Bray curtis matrix, the PS group tended to be located between the YN and ON groups based on PC 1. In Jaccard, the plot tendency moves to the right in the order of YN, ON and PS groups based on PC 1.

**Fig. 3:**
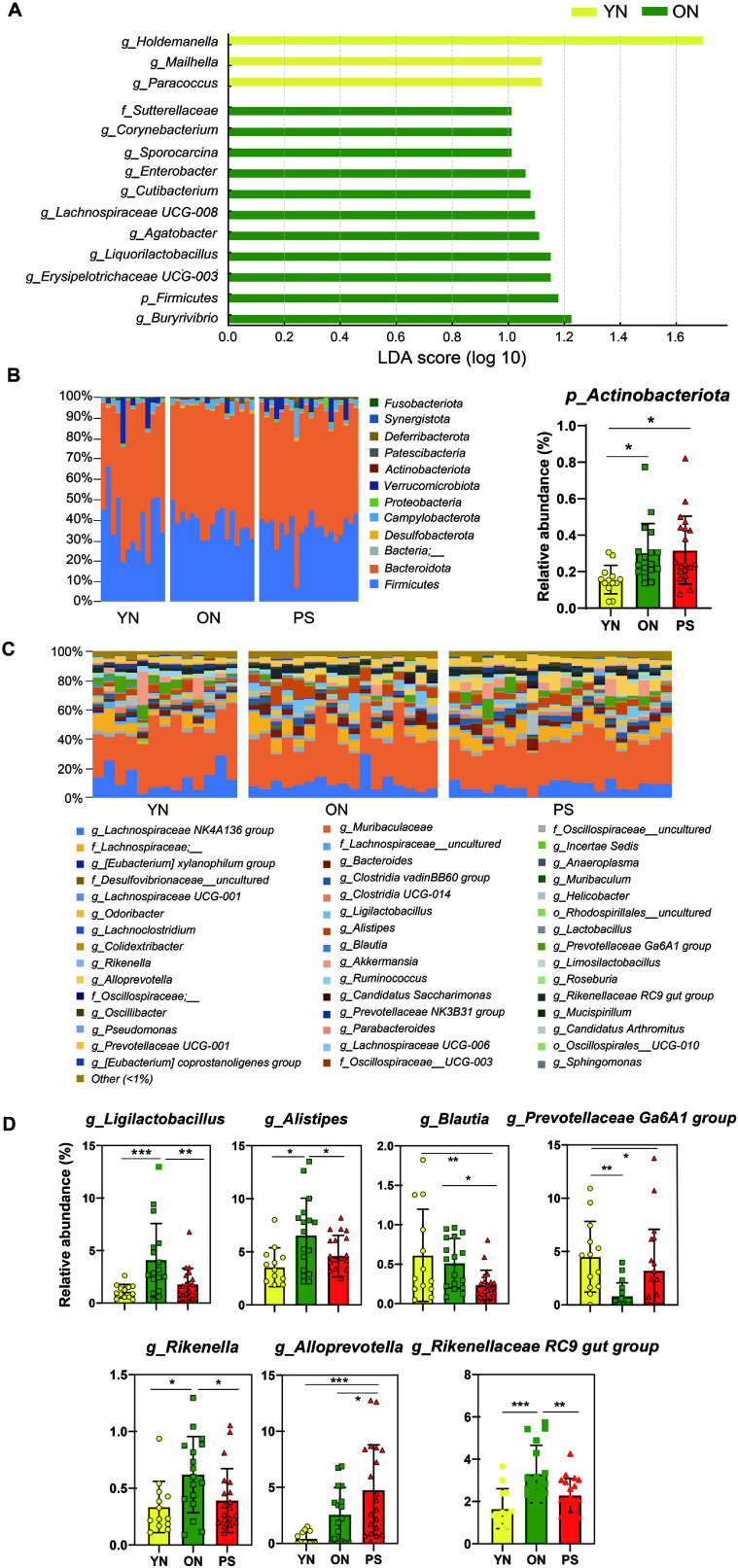
Relative abundances of gut microbiota and LDA in YN, ON and PS groups. Bar graphs of the LDA score based on LefSe analysis (**A**). Bacterial composition at the phylum (**B**), and genus (**D**) levels. Relative abundance of microbiota at the phylum (**C**) and genus level showing significant differences among the three groups (YN, ON and PS) (**E**). Data are expressed as mean ± SD. * *p* < 0.05.

The average relative abundance of the gut microbiome at the phylum and genus levels in average relative abundance of gut microbiome is shown in Fig. 3A. At the phylum and genus level, Lefse detected 14 bacterial clades, which showed statistically significant differences between groups with linear discriminant analysis (LDA) scores higher than 1. *Holdemanella*, *Mailhela* and *Paracoccus* were detected as the most powerful markers in YN groups. *Sutterellaceae*, *Corynebacterium*, *Sporosarcina*, *Enterobacter*, *Cutibacterium*, *Lachnospiraceae UCG-008*, *Agathobacter*, *Liquorilactobacillus*, *Erysipelotrichaceae UCG-003*, *Firmicutes* and *Butyrivibrio* were detected as markers in the ON groups. No bacterial taxa with LDA score greater than 1 were observed in the ON and PS groups (Data not shown).

As shown in Fig. 3B and 3C, the gut microbiota composition was shown at the phylum and genus levels. At the phylum level, *Firmicutes* and *Bacteroidota* were the predominant phyla. In the PS groups, *Actinobacteriota* had a relatively higher abundance compared to the other groups (YN, ON) (Fig. 3B). At the genus level, the top 5 bacteria were *Lachnospiraceae NK4A136* group, *Muribaculaceae*, *Oscillospiraceae*_uncultured, *Lachnospiraceae*; __, and *Lachnospiraceae*;__uncultured (Fig. 3C). Seven strains (*Ligilactobacillus*, *Alistipes*, *Blautia*, *Prevotellaceae Ga6A1 group*, *Rikenella*, *Alloprevotella* and *Rikenellaceae RC9* gut group) showed significant differences between the three groups (YN, ON and PS), *Blautia* showed a significantly decreased in order to YN, ON, and PS. Whereas *Alloprevotella* showed a significantly increased in the opposite direction (Fig. 3D).

### Effects of PS Treated Group on Fecal Metabolites

**Fig. 4:**
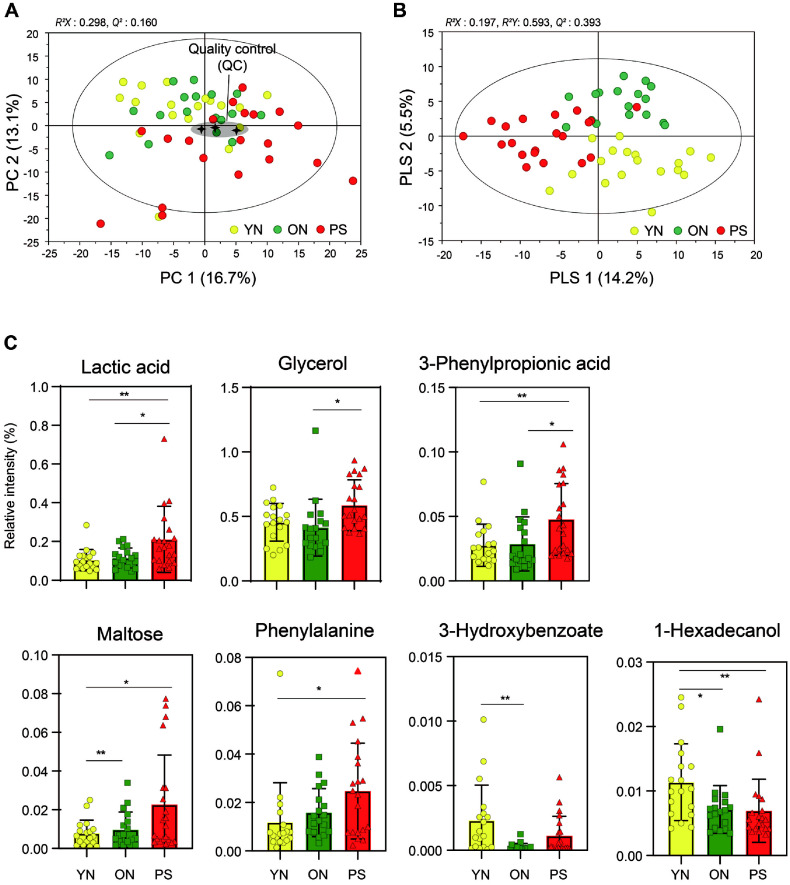
Fecal metabolites were altered by control (YN, ON) and PS treatment group. Principal-component analysis (PCA) plot (**A**) and partial least squares discriminant analysis (PLS-DA) score plot (**B**) of fecal metabolites in three groups. Relative abundance of metabolites showing significant differences among the three groups (YN, ON and PS) (**C**). Data are expressed as mean ± SD. Selected metabolites identified with a VIP score of 1.0 and significantly different metabolites are marked with * *p* < 0.05.

A total of 50 metabolites were identified (Table S1). These metabolites included various biomolecules such as amino acids, organic acids, sugars, and fatty acids. These metabolites were major contributors to differences between the YN, ON, and PS groups. The results of the principal-component analysis (PCA) showed no significantly difference between groups (Fig. 4A). The plot of partial least squares discriminant analysis (PLS-DA) revealed that the composition of fecal metabolites separated in order to PS, ON, and YN group from the left based on PC 1 (Fig. 4B). Five metabolites (Lactic acid, Glycerol, 3-Phynylpropionic acid, Maltose and Phenylalanine) showed a tendency to be significantly increased in the PS group. While 1-Hezadecanol showed a tendency to decrease in the PS group compared to the YN and ON groups (Fig. 4C).

### Polystyrene Treatment Changed the Profile of Fecal Metabolites

**Fig. 5:**
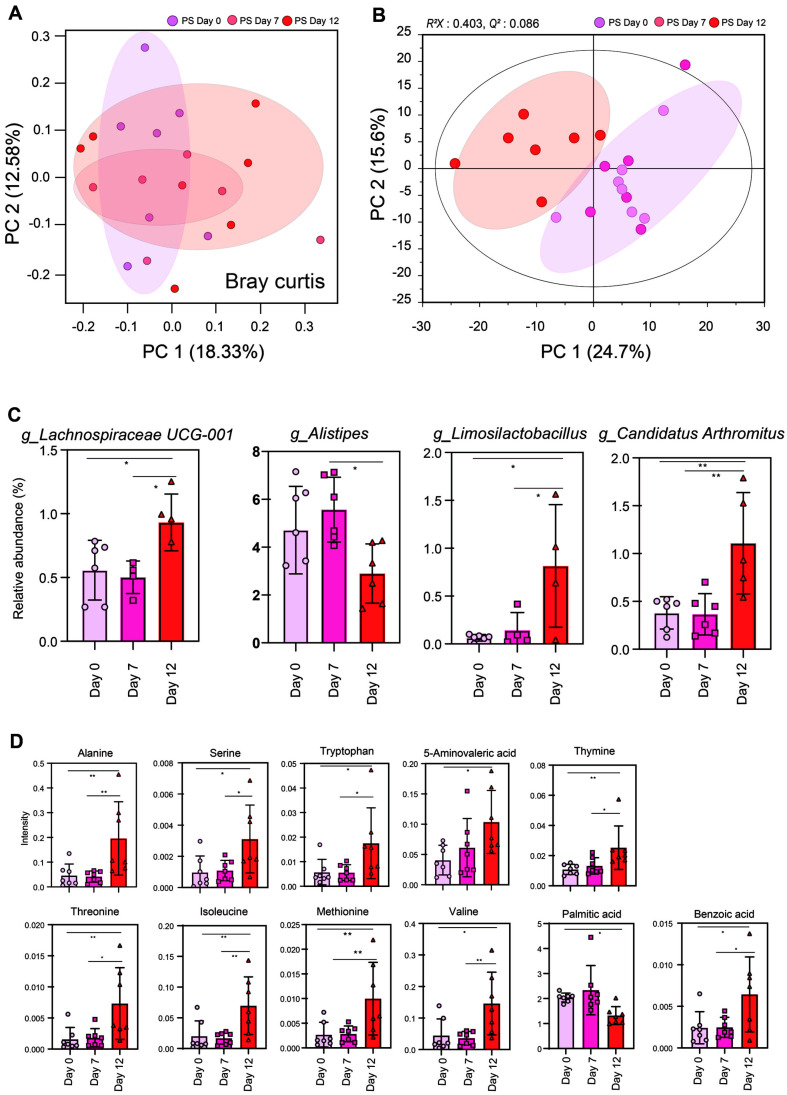
Fecal microbiome and metabolite were altered by PS treatment group of administration date. Principal-component analysis (PCA) plot of Bray curtis of fecal microbiome (**A**) Principal-component analysis (PCA) plot (**B**) of fecal metabolites in three groups. (**C**) Microbial taxa and (**D**) metabolite that exhibited an increasing or decreasing trend following PS administration. Data are expressed as mean ± SD. Selected metabolites identified with a VIP score of 1.0 and significantly different metabolites are marked with * *p* < 0.05.

To examine the changes in gut microbiota and fecal metabolites following PS administration, a PCA score plot was analyzed (Fig. 5A and 5B). The results showed that the microbiota and metabolite plots exhibited a leftward shift along the PC1 axis with PS administration.

The analysis of microbial changes in response to PS exposure revealed a tendency toward increases in *Lachnospiraceae UCG-001*, *Limosilactobacillus and Candidatus Arthromitus* whereas *Alistipes* showed a decreasing tendency (Fig. 5C). In the case of metabolites, all detected metabolites (alanine, serine, tryptophan, 5-aminovaleric acid, thymine, threonine, isoleucine, methionine, valine, and benzoic acid) except palmitic acid tended to increase over the administration period (Fig. 5D).

### Correlation Analysis of Microbial Genera and Fecal Metabolite

Spearman’s rank correlation was calculated to explore the potential functional relationships between gut microbiome and metabolites. As shown in Fig. S1, Lactic acid, Lyxose, Thymine, Fucose and Isoleucine were positively correlated with Serine, Tryptophan, Alanine. Tryptophan was positively correlated with and *Alloprevotella* (r = 0.71). Arachidonic acid was positively correlated with *Rikenella* (r = 0.7).

### Enrichment Analysis of Differential Metabolites

**Fig. 6:**
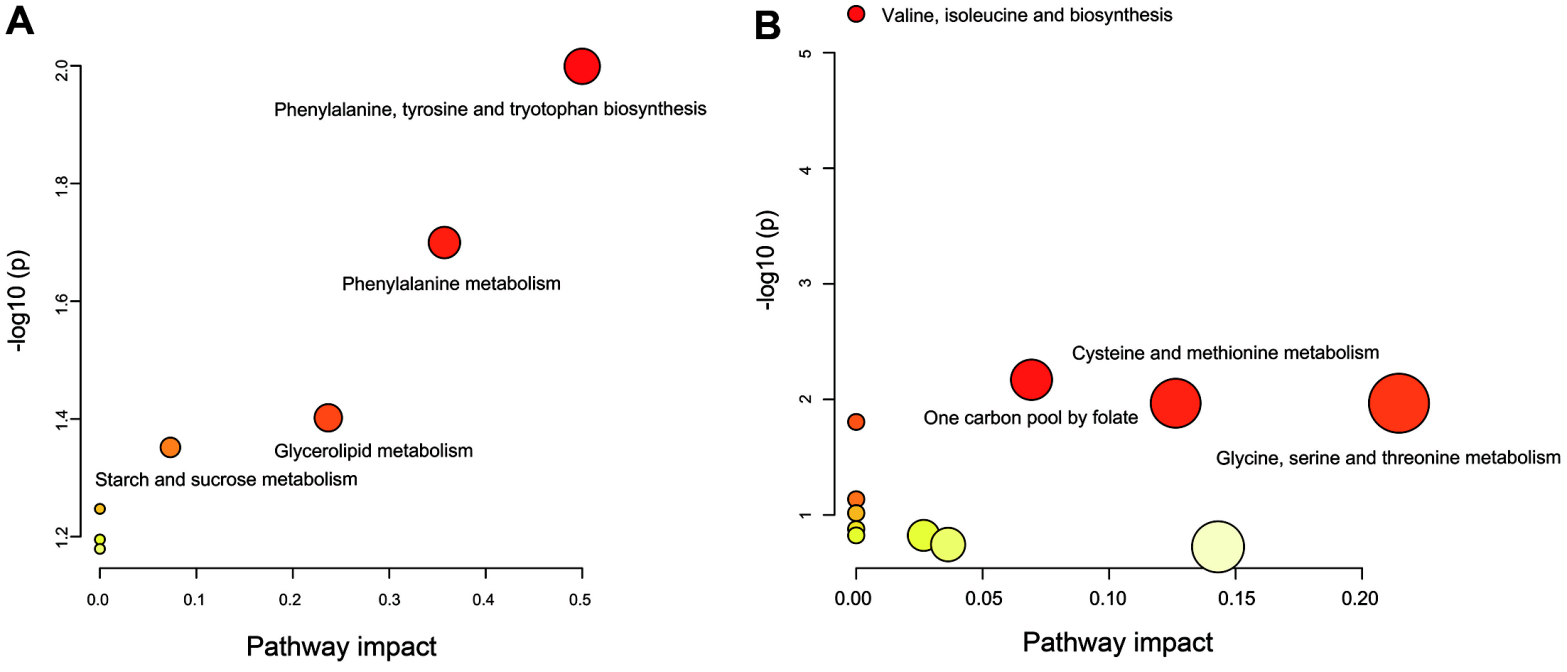
Summary of pathway analysis by MetaboAnalyst 6.0. The top pathways are ranked based on gamma-adjusted *p*-values for permutation per pathway (y-axis) and the total number of occurrences per pathway (x-axis). The color gradient transitions from white to yellow, orange, and red as both x and y values increase. (**A**) Metabolic pathways that show significant changes among the three groups (**YN, ON, PS**). (**B**) Metabolic pathways significantly altered during the PS administration period.

In order to further understand the usefulness of the detected metabolites by the GC-MS method, we performed pathway analysis to associate the metabolites to their corresponding pathways (Fig. 6). The results of the enrichment analysis showed that Phenylalanine, tyrosine and tryptophan biosynthesis; Phenylalanine metabolism; Glycerolipid metabolism; and Starch and sucrose metabolism were significantly enriched (*p* < 0.05) in the YN, ON and PS groups. Pathways valine, leucine and isoleucine biosynthesis; One carbon pool by folate; Cysteine and methionine metabolism; Glycine, serine and threonine metabolism; and Valine, leucine and isoleucine degradation were significantly enriched (*p* < 0.05) on day 0, 7 and 13 in the PS groups.

## Discussion

Nano-sized PS plastics is readily absorbed and accumulated in human organs [23]. We presumed that PS exposure might accelerate aging. In the PS-treated group, spleen and kidney weights tend to increase compared to the YN and ON groups (Fig. 1F and 1G). Importantly, PS can also accumulate in human intestines and may pass through the intestinal barrier to the immune system [24]. In ALT levels, a marker of liver and kidney toxicity [25], the PS treatment group showed a tendency to increase compared to the ON group. ALT is an important indicator of liver function and is closely related to liver diseases such as liver fibrosis [26]. In this result, liver weight increased in the ON and PS groups compared to the YN group (Fig. 1E). Serum alanine aminotransferase (ALT) activity has been widely utilized as a key biomarker for evaluating liver inflammation and injury associated with various hepatic disorders, including hepatitis, liver malignancies, and cirrhosis. However, the ALT/AST ratio is considered a more reliable indicator for distinguishing different types of liver damage and assessing disease severity [27]. Increases in ALT are commonly observed in liver and muscle disorders, likely due to the release of this enzyme into the bloodstream as a consequence of cellular damage [28]. As aging progresses, changes in gut microbiome due to PS exposure can become more rapid including cellular damage. We confirmed that the PS group had changes in gut microorganisms and fecal metabolites compared to the ON group.

In gut microorganism composition is changed by mucous membrane cells. In this study, mice fed PS microplastics showed that *Blautia* was lower than YN group and *Alloprevotella* was higher than in the YN group. The genus *Blautia* comprises anaerobic bacteria with probiotic properties, and is commonly found in the intestines and feces of mammals [29]. *Blautia* has been reported to contribute to the regulation of energy metabolism and host inflammatory responses. According to a study by Mao *et al*. (2024) [29] the administration of *Blautia producta* in a DSS-induced model led to the suppression of inflammatory cytokine elevation, including interleukin-6 (IL-6), tumor necrosis factor-α (TNF-α), and interleukin-1β (IL-1β), as well as a reduction in excessive oxidative stress, as indicated by changes in myeloperoxidase (MPO) and superoxide dismutase (SOD) activity [30]. Furthermore, the abundance of *Blautia* has been reported to decrease with aging [31], suggesting its potential as a biomarker related to aging and immune function. *Alloprevotella* is genus level bacterium in the *Prevotellaceae* family and has been suggested to have both positive and negative effects on health. Yao *et al*. (2021) [32] reported that *Alloprevotella* is a genus level that produces short-chain fatty acids, especially butyric acid, that can maintain intestinal homeostasis. However, other study reported that increased abundance of *Alloprevotella* has been observed in aged female mice with irritable bowel syndrome (IBS) [33]. Additionally, *Alloprevotella* has been identified as a pathogen associated with chemotherapy-induced diarrhea in mice [34]. While numerous studies have reported the association of this strain (*Alloprevotella*) with human immunity, further research is required to elucidate whether it plays a more prominent role in disease treatment or disease induction. Due to maintain gut microbiome homeostasis and health of the host, the *Lachnospiraceae* family plays an important role. *Lachnospiraceae UCG-001* showed high relative abundance over the PS administration period (Fig. 5C). This strain (*Lachnospiraceae*) is closely related to disease. Lu *et al*. (2021) [35] reported that an analysis of microbial diversity in the feces of mice with active ulcerative colitis (UC) revealed that *Lachnospiraceae UCG-001* exhibited the significant changes in relative abundance. Other reported that the abundance of *Lachnospiraceae* may be influenced by alterations in lipid metabolism and inflammatory processes, potentially contributing to intestinal microbiota imbalance in aged C57BL/6J mice [36]. These findings suggest that gut microbiota alterations induced by PS consumption may serve as potential biomarkers associated with various diseases and could further contribute to the acceleration of aging.

Fecal metabolites play important roles in gut health and homeostasis. Phenylalanine is an essential amino acid that must be obtained from the diet, as it cannot be endogenously synthesized in the human body. Elevated levels of phenylalanine have been observed in patients with contact dermatitis and infants with food allergies [37]. Excessive accumulation of phenylalanine in the body leads to the production of various toxic metabolites. Dysregulation of phenylalanine metabolism has been reported in the human hippocampus and is associated with the pathological changes observed in Alzheimer's disease (AD). Moreover, studies have indicated that patients with phenylketonuria (PKU), a rare disorder characterized by cranial nerve damage due to abnormal phenylacetone metabolism, exhibit elevated levels of Aβ1-42 and tau in the cerebrospinal fluid (CSF) [38]. Thymine is one of the bases that make up DNA. It produces a by-product (thymidine glycol), which is detected after oxidation or irradiation of DNA *in vitro* and *in vivo* such as Alzheimer’s disease [39] and liver tumor [40, 41]. This metabolite is known to increase as aging progresses, and Paley *et al*. [42] study confirmed that Alzheimer's patients' plasma thymine content increased compared to the normal group. In this study, Thymine levels exhibited an increasing trend following PS administration (Fig. 5D). Palmitic acid was significantly decreased in the PS group after administration (Fig. 5D). Palmitic acid, the most common saturated fatty acid in plant and animal species, has important biological functions such as energy storage and maintaining cell membrane integrity [43]. In the Delmonico study [44], when analyzed using GC-MS, palmitic acid was found to be decreased in the sarcopenia and mild cognitive impairment groups compared to the normal group. Studies have shown that palmitic acid can promote the release of large amounts of inflammatory cytokines including interleukin-6 (IL-6) [45], IL-8 [46] and NF-kB [47, 48]. Inflammation is one of the important pathological factors that determines the acceleration of aging. Additional studies are needed to investigate whether low levels of palmitic acid are associated with the relevant pathways of cytokine oxidative stress that cause aging.

In conclusion, our study confirmed that PS administration induces gut microbiota dysbiosis, which may contribute to the acceleration of aging. Our research is one of the few studies suggesting the potential role of PS in promoting aging. However, a limitation of this study is the short 4-week exposure period. While chronic exposure is typically needed to assess long-term aging effects, short-term PS exposure can still induce significant changes, including oxidative stress and gut microbiota alterations [49, 50]. Additionally, the lack of a young PS-treated group limits our ability to fully assess aging acceleration. Future studies will include a young PS-treated group to better understand its effects on aging.

## Supplemental Materials

Supplementary data for this paper are available on-line only at http://jmb.or.kr.
