# Supplementary material for: Polystyrene Accelerates Aging Related-Gut Microbiome Dysbiosis and -Metabolites in Old-Aged Mouse
Source: J Microbiol Biotechnol. 2025 Aug 7;35:e2504016. doi: 10.4014/jmb.2504.04016 (PMC12351109; doi:10.4014/jmb.2504.04016)
Supplement: Supplementary file 1 [file jmb-35-e2504016-supple.pdf]

J. Microbiol. Biotechnol. 2025. 35: e2504016

https://doi.org/10.4014/jmb.2504.04016

Supplementary Table

Table S1. Identified metabolites In fecal samples and their changes during PS treatment.

No.	Metabolites	Retention time (min)	Target Mass	Average retention index (RI)
1	Propyleneglycol	4.503	117.1	1003.91
2	Lactic acid	5.230	117.1	1063.81
3	Alanine	5.750	116.1	1106.85
4	beta-Hydroxybutyric acid	6.415	147.05	1163.98
5	Valine	7.090	144.1	1223.40
6	Urea	7.249	147.0583	1237.96
7	Benzoic acid	7.420	179.0156	1253.71
8	2-Aminoethanol	7.661	174.1	1275.76
9	Glycerol	7.719	147.05	1281.07
10	Phosphate	7.730	299.0526	1282.15
11	Nicotinic acid	7.917	180	1299.29
12	Isoleucine	7.940	158.1019	1301.48
13	Glycine	8.090	147.05	1316.41
14	Uracil	8.399	241.05	1347.21
15	Serine	8.618	204.1	1368.99
16	Threonine	8.903	219.0955	1397.29
17	Thymine	9.035	255.0535	1411.15
18	3-Phenylpropionic acid	9.179	104.0975	1426.45
19	Threitol	10.059	217.0786	1521.53
20	Methionine	10.147	176.0981	1531.48
21	4-Aminobutyric acid	10.243	174.1	1542.38
22	3-Hydroxybenzoate	10.549	267.0555	1577.17
23	Glutamic acid	10.986	246.1032	1628.41
24	5-Aminovaleric acid	11.090	174.1	1641
25	Phenylalanine	11.120	218.05	1644.56
26	4-Hydroxyphenylacetic acid	11.156	252.1046	1648.92
27	Xylose	11.388	103.0871	1676.87
28	Lyxose	11.441	103.0919	1683.23
29	Ribose	11.565	103.0789	1698.15
30	Indoxyl sulfate	11.826	277.1	1731.31
31	Rhamnose	12.001	160.075	1753.65
32	Fucose	12.119	117.1	1768.60
33	Hypoxanthine	12.550	265.0645	1824.63

34	Myristic acid	12.744	117.05	1850.56
35	Fructose	13.123	103.0929	1901.09
36	Glucose	13.330	319.15	1930.28
37	Mannose	13.479	319.1424	1951.22
38	Tyrosine	13.533	218.0546	1958.84
39	1-Hexadecanol	13.544	299.2588	1960.46
40	Pantothenate	13.923	103.0893	2014.55
41	Xanthine	14.105	353.1	2041.48
42	Plamitic acid	14.150	117.05	2048.21
43	N-Acetyl galactosamine	14.747	202.0982	2137.97
44	Elaidic acid	15.296	117.05	2223.58
45	Stearic acid	15.433	117.05	2245.69
46	Tryptophan	15.494	202.0922	2255.56
47	arachidonic acid	16.266	91.08864	2383.35
48	Maltose	18.593	361.1483	2814.61
49	Melibiose	19.251	361.1437	2944.42
50	α-Tocopherol	20.646	237.1	3190.32
